# Supplementary material for: Stress sensitization among severely neglected children and protection by social enrichment
Source: Nat Commun. 2019 Dec 18;10:5771. doi: 10.1038/s41467-019-13622-3 (PMC6920417; doi:10.1038/s41467-019-13622-3)
Supplement: Supplementary file 1 — Supplementary Information [file 41467_2019_13622_MOESM1_ESM.pdf]

Stress sensitization among severely neglected children and protection by social enrichment: Follow-up of a randomized controlled trial

Wade et al.

Supplementary Materials

Supplementary Methods

BEIP Life Events Scale

Please note whether any of these life events have happened to you or your family in the past 12 months. If yes, please indicate how stressful the event was for you and the extent of control you believe you had over the event.

IN THE PAST 12 MONTHS…	Did this happen?		I/D
1. You began dating.	NO	YES	D
2. You changed schools.	NO	YES	I
3. You started wearing glasses, contacts, or braces.	NO	YES	I
4. You failed a grade in school or got bad grades.	NO	YES	D
5. You got into serious trouble at school (e.g., suspended).	NO	YES	D
6. You did not make an extracurricular activity you wanted to be involved in.	NO	YES	D
7. You and your boyfriend/girlfriend had a big fight or broke up.	NO	YES	D
8. A good friend moved away.	NO	YES	I
9. Your family moved to a new home or apartment.	NO	YES	I
10. You had to start sharing a bedroom with someone or no longer have a bedroom.	NO	YES	I
11. A family member moved in or out of the house or ran away (e.g., brother or sister went into army, started college, or left home; grandparents moved in).	NO	YES	I
12. You went to live with your other parent or another relative.	NO	YES	I
13. Your family's house or car was broken into or robbed.	NO	YES	I
14. A family member was attacked or robbed.	NO	YES	I
15. You were attacked or robbed.	NO	YES	I
16. A family member who lives with you (e.g., mother, aunt, sister) got pregnant, had a baby, or had a difficult pregnancy.	NO	YES	I
17. Your family had less money for important things (e.g., food, electricity, rent, etc.) or had serious money problems (e.g., bankruptcy, went on welfare, possessions taken away).	NO	YES	I
18. A family pet died or was lost.	NO	YES	I
19. You had a serious accident or illness and were in the hospital.	NO	YES	I
20. A family member started having serious health problems, was in an accident, or went into the hospital or nursing home.	NO	YES	I
21. A family member or close relative died.	NO	YES	I
22. A close friend had a serious illness or injury or died.	NO	YES	I
23. Your parents separated or got divorced.	NO	YES	I
24. You saw a parent much less than you used to.	NO	YES	I
25. A parent got engaged or remarried, so you got a new stepfather, stepmother, stepsister(s) or stepbrother(s).	NO	YES	I
26. There were more arguments or fights between your parents.	NO	YES	I
27. You had more arguments or fights with one of your parents.	NO	YES	D
28. A family member got in trouble with the law, was arrested, or went to prison.	NO	YES	I
29. You got in trouble with the police (arrested, went to court, etc.).	NO	YES	D
30. Other ___________________________	NO	YES	-

This scale was adapted from Coddington (1972)

Note. I – independent event (an event over which the child has virtually no control); D – dependent event (an event over which the child has at least some degree of control).

Supplementary Results

Supplementary Table 1: Comparison of institutionalized children who remained in the sample and those who dropped out at some point up to age 16 (*N* = 136)

	Still in Study (n = 109)		Dropped Out (n = 27)		Difference
	M or %	*SD*	*M*	*SD*	*p*-value
Age at beginning of baseline evaluation	20.52	7.23	19.95	7.82	.72
Age at the end of the baseline evaluation	21.21	7.20	20.73	8.06	.77
Birth weight	2792.19	598.30	2704.62	610.60	.51
Gestational age	37.12	2.22	37.87	1.79	.14
Gender					
Male	51.4% (56/109)		40.7% (11/27)		.32
Female	48.6% (53/109)		59.3% (16/27		
Ethnicity					
Romanian	51.4% (56/109)		74.1% (20/27)		.03
Other	48.6% (53/109)		25.9% (7/27)		
Diagnosis of developmental disability					
No	95.4% (104/109)		100% (27/27)		.26
Yes	4.6% (5/109)		0% (0/27)		

Note. The only difference between those who remained in the study versus those who dropped out was on Ethnicity, with those who dropped out being more likely to be Romanian versus “other”.

Group Differences in Life Events and Relation to Caregiving Quality

As seen in Supplementary Figure 1 and reported in the main text, the care-as-usual children reported more total life events than both the foster care and never-institutionalized children, as well as more dependent life events than the foster care children, suggesting a modest intervention effect on the experience of these events. The difference between the foster care and care-as-usual children may be due to their responsiveness to caregiving experiences during (pre)adolescence, with the foster care children experiencing higher quality care and being buffered against the experience of stressful events to a greater degree than the care-as-usual children. Indeed, using a measure of caregiving quality, we see that the foster care group experience higher quality caregiving at age 12 and 16 years relative to the care-as-usual children ($t = 3.82$, $p < .001$ at age 12; $t = 3.14$, $p = .002$ at age 16). Moreover, using Poisson regression we see that, over and above the effects of group (foster care vs. care-as-usual), higher-quality caregiving is associated with lower total (wald $\chi^2 = 15.09$, $p < .001$), dependent (wald $\chi^2 = 10.03$, $p = .002$), and independent (wald $\chi^2 = 8.18$, $p = .004$) life events. Thus, the foster care children experience higher quality care, on average, compared to the care-as-usual children, and this higher-quality care is related to fewer life events.

Finally, using Poisson regression, we see that the effect of caregiving quality on stressful life events may be stronger for the foster care versus the care-as-usual children. Specifically, while higher quality caregiving is associated with fewer total life events among the care-as-usual children (wald $\chi^2 = 4.39$, $p = .04$), this effect is even stronger among the foster care children (wald $\chi^2 = 13.03$, $p < .001$). We see the same pattern for dependent life events (care-as-usual: wald $\chi^2 = 2.97$, $p = .085$; foster care: wald $\chi^2 = 8.61$, $p = .003$) and independent life events (care-as-usual: wald $\chi^2 = 2.99$, $p = .084$; foster care: wald $\chi^2 = 5.81$, $p = .016$). From these results, it appears that the difference between the foster care and care-as-usual children is larger for dependent versus independent events. Thus, not only do the foster care children experience higher quality care, but higher quality care may exert a stronger protective effect against stressful life events for the foster care children compared to the care-as-usual-children.

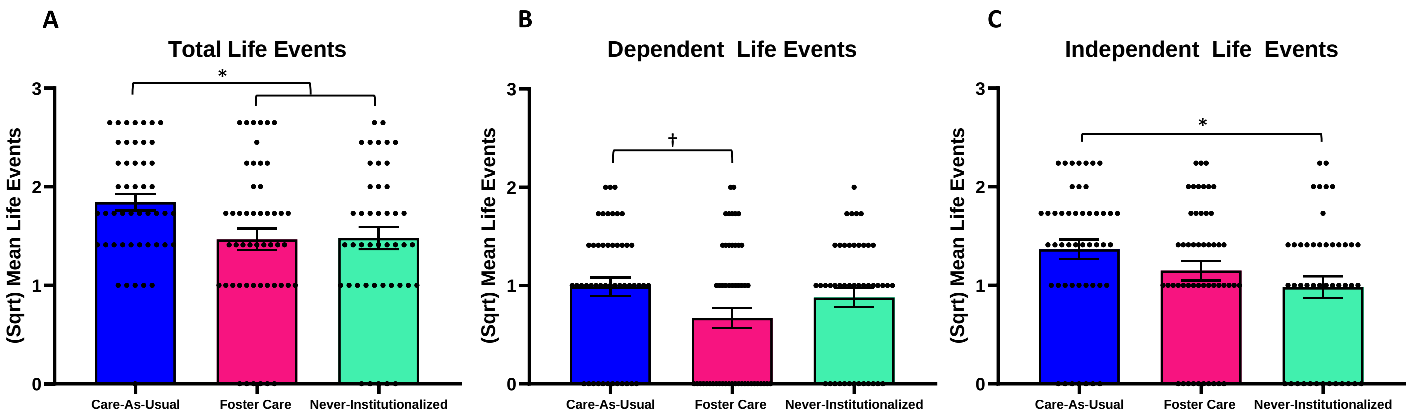

Supplementary Figure 1: Group Differences in Life Events at Age 12
Mean differences between the care-as-usual, foster care, and never-institutionalized groups on the number of total (A), dependent (B), and independent (C) life events at age 12, evaluated using ANOVA. For this analysis only, the life events variable was square-root transformed to account for the right-skew of the of life events measure. In the primary regression model, the linear variable, scored as 0 to 7+ events, was used. Pairwise group differences are planned comparisons with no adjustment. Error bars are standard errors. $^{*}p < .05$. $^{\dagger}p < .10$.

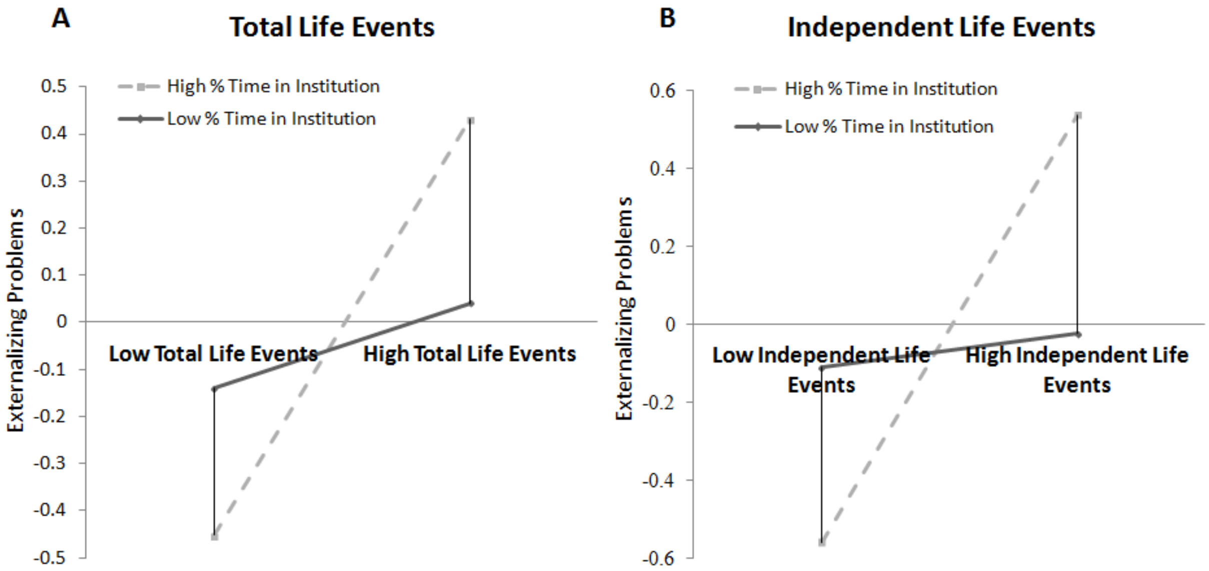

Supplementary Figure 2: Interaction Between Stressful Life Events and Time Spent in Institutions on Externalizing Problems at Age 16.
The effect of total (A) and independent (B) life events on externalizing problems as a function of the percentage of one's life spent in an institution among foster care and care-as-usual children. Effects are plotted +/- 1 *SD* above and below the mean. As the number of life events increased, externalizing problems increased as time spent in institutions increased up to age 16.

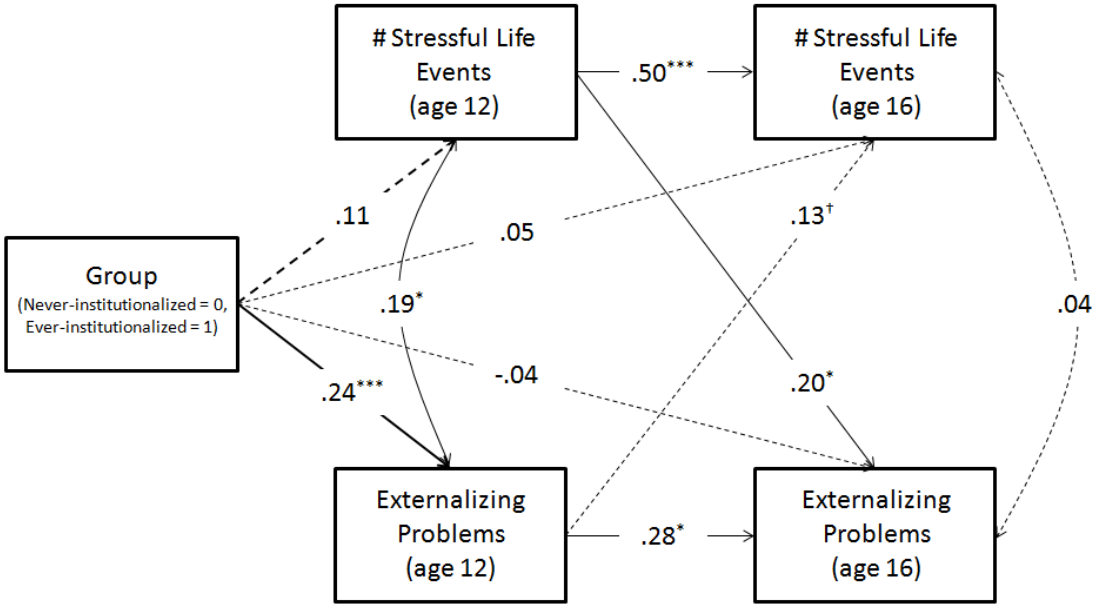

Supplementary Figure 3: Reciprocal Relations between Stressful Life Events and Externalizing Problems from Age 12 to Age 16

Results from the cross-lagged longitudinal model with stability coefficients and within-time covariance terms included. Institutionalization status and gender (not shown) were also included in the model. The number of stressful life events at age 12 positively predicted externalizing problems at age 16, yet the reverse effect of externalizing problems at age 12 predicting life events at age 16 was only marginal. Thus, after accounting for group and gender, there appears to be a directional link between life events and externalizing problems, supporting our primary finding that more life events increase susceptibility to externalizing problems in the context of prolonged institutional rearing. $^{***}p < .001$. $^{**}p < .01$. $^{*}p < .05$. $^{\dagger}p < .10$.

Alternative Model: Stress Generation

Background of Stress Generation Model

An alternative hypothesis to stress sensitization is that childhood adversity is generative of later stressful life events that increase the risk of psychopathology. It has been shown that continued stress exposure proximal to psychopathology accounts for the association between adversity in childhood and psychiatric problems in adolescence.[1] The extent of childhood adversity has been shown to relate to the number of stressful life events in a dose-response manner.[2] On this basis, children experiencing prolonged institutional rearing would be expected to experience more stressful life events later in development than those removed from institutions early and placed into foster care. Moreover, in contrast to the stress sensitization hypothesis, this "stress generation" hypothesis may manifest more clearly for *dependent* compared to independent life events. For instance, childhood adversity may impact the development of biobehavioral systems which give rise to dysregulated patterns of cognition and behavior, in turn contributing to the occurrence of other stressful events such as school suspensions, getting in trouble with the law, or more frequent arguments with caregivers. In support of this hypothesis, the link between childhood adversity and later stressful life events is mediated by individual personality characteristics.[3,4] Among children with histories of institutional rearing, externalizing problems in early adolescence relate to traits such as higher sensation-seeking.[5] Institutionally-reared children also tend to have poorer executive functioning,[6] and to be more mistrusting than never-institutionalized children.[7] These phenotypes may heighten the risk of externalizing problems by increasing the number of dependent life events. Thus, as an alternative to stress sensitization, we also tested whether severe childhood neglect was indirectly associated with externalizing problems in adolescence through stressful life events in preadolescence.

Statistical Analysis for Stress Generation Model

The association between group (ever- versus never-institutionalized; care-as-usual versus foster care) and stressful life events was examined using Generalized Linear Models to account for the significant right-skew of the life events variable (this is the *a* path in Supplementary Figure 4). Second, the relation between stressful life events

and externalizing problems was examined using linear regression (*b* path in Supplementary Figure 4). Finally, the indirect effect of group on externalizing problems through life events was assessed in Mplus using boostrapping to determine the significance of the indirect path.

Results of Stress Generation Model

We tested the alternative hypothesis that childhood neglect was associated with externalizing problems at age 16 through stressful life events at age 12. First, controlling for gender, Poisson regression revealed that institutionalization was not related to the number of total life events, $B = .16$, 95% CI [-.05, .37], wald $\chi^2 = 2.31$, $p = .13$, or dependent events, $B = .01$, 95% CI [-.32, .33], wald $\chi^2 < .01$, $p = .98$, but was related to the number of independent events, $B = .36$, 95% CI [.08, .63], wald $\chi^2 = 6.31$, $p = .012$. Among previously institutionalized children, being in the foster care group was associated with significantly fewer total life events compared to the care-as-usual group, $B = -.31$, 95% CI [-.53, -.08], wald $\chi^2 = 7.14$, $p = .008$. This effect was significant when the analysis was limited to dependent events, $B = -.38$, 95% CI [-.75, -.01], wald $\chi^2 = 4.13$, $p = .042$, but only marginally so when limited to independent events, $B = -.24$, 95% CI [-.51, .03], wald $\chi^2 = 2.95$, $p = .09$. Thus, consistent with results from Supplementary Figure 1, belonging to the foster care group was associated with fewer total life events at age 12 compared to care-as-usual group, and this effect was stronger for dependent than independent events (Supplementary Figure 4).

Second, for the *b* path, total life events at age 12 was positively associated with externalizing problems at age 16, $\beta = .20$, 95% CI [.04, .35], $p = .013$ after controlling for gender and externalizing problems at age 12. This effect was not significant for dependent events, $\beta = .09$, 95% CI [-.08, .24], $p = .31$, but was significant for independent events, $\beta = .25$, 95% CI [.09, .39], $p = .002$. Among previously institutionalized children, the same pattern was observed: total life events was positively associated with externalizing problems, $\beta = .21$, 95% CI [.02, .44], $p = .030$. This effect was not significant for dependent events, $\beta = .07$, 95% CI [-.14, .29], $p = .48$, but was significant for independent events, $\beta = .28$, 95% CI [.10, .50], $p = .004$. Thus, independent, but not dependent, life events predicted higher externalizing problems in adolescence.

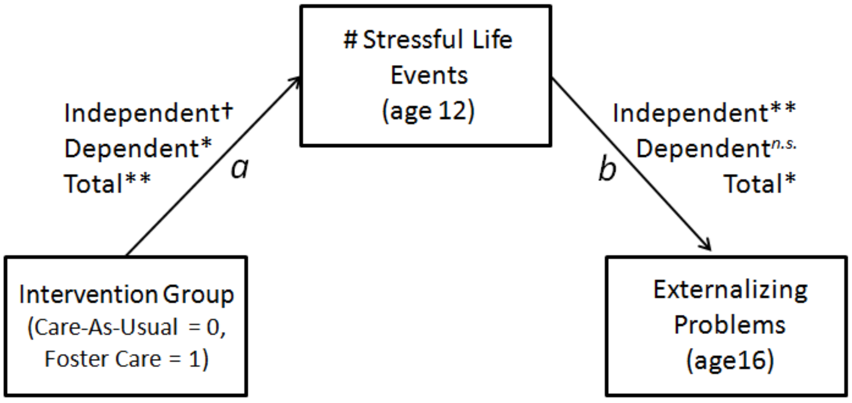

Supplementary Figure 4: Alternative Model – Stress Generation

Effect of (*a*) the intervention on the number of stressful life events at age 12; and (*b*) stressful life events on externalizing problems at age 16. Significance values for total, dependent, and independent life events are shown (exact statistics are presented in-text).$^{**}p < .01$. $^{*}p < .05$. $^{\dagger}p < .10$. *n.s.* – not significant. None of the indirect effects of group on externalizing problems through life events were significant.

Finally, after controlling for gender and externalizing problems at age 12, no significant indirect effects were observed, either for the effect of institutionalization or foster care intervention on externalizing problems through life events (all *p*'s > .10). This was the case whether total, dependent, or independent events were used in the analysis. Thus, little support was found for the stress generation model.

Discussion of Stress Generation Model

Overall, little support was found for the hypothesis that profound early neglect was generative of later stressful life events that increased the risk of externalizing problems in adolescence. There was suggestive, though comparatively weak, evidence that children assigned to early foster care had lower future risk of experiencing

stressful life events compared to those with prolonged institutional rearing. This was especially true of dependent events. This finding, while requiring validation in studies with larger samples, is consistent with the idea that ongoing exposure to adversity may be generative of future stressful events. Indeed, characteristics of individuals that are shaped by early adversity may account for increases in dependent life events proximal to psychopathology, such as negative cognitive style and executive dysfunction.[8,9] However, the number of dependent events did not predict externalizing problems in the current sample, and as a result there were no indirect effects from early neglect or foster care intervention to externalizing problems via dependent events. Indeed, none of the indirect effects involving total, dependent, or independent events emerged as significant mediators of on externalizing problems in adolescence in the current study. It is possible that our analysis was underpowered to detect such effects, especially the difference between the care-as-usual and foster care groups, for which the sample size was modest. Thus, we found stronger support for stress sensitization than stress generation in predicting externalizing problems. Nonetheless, future studies with larger samples are warranted to determine whether childhood adversity sensitizes individuals to later independent events, while also predisposing them to more dependent events.

Supplementary References

1. Hazel N, Hammen C, Brennan P, Najman J. Early childhood adversity and adolescent depression: the mediating role of continued stress. *Psychological Medicine* 2008; **38**(4): 581-9.
2. Korkeila J, Vahtera J, Nabi H, et al. Childhood adversities, adulthood life events and depression. *Journal of affective disorders* 2010; **127**(1): 130-8.
3. Hovens JG, Giltay EJ, Hemert AM, Penninx BW. Childhood maltreatment and the course of depressive and anxiety disorders: the contribution of personality characteristics. *Depression and anxiety* 2016; **33**(1): 27-34.
4. Hayashi Y, Okamoto Y, Takagaki K, et al. Direct and indirect influences of childhood abuse on depression symptoms in patients with major depressive disorder. *BMC psychiatry* 2015; **15**(1): 244.
5. Loman MM, Johnson AE, Quevedo K, Lafavor TL, Gunnar MR. Risk-taking and sensation-seeking propensity in postinstitutionalized early adolescents. *Journal of child psychology and psychiatry* 2014; **55**(10): 1145-52.
6. Bick J, Zeanah CH, Fox NA, Nelson CA. Memory and Executive Functioning in 12-Year-Old Children With a History of Institutional Rearing. *Child development* 2018; **89**(2): 495-508.
7. Pitula CE, Wenner JA, Gunnar MR, Thomas KM. To trust or not to trust: social decision-making in post-institutionalized, internationally adopted youth. *Developmental science* 2017; **20**(3).
8. Safford SM, Alloy LB, Abramson LY, Crossfield AG. Negative cognitive style as a predictor of negative life events in depression-prone individuals: A test of the stress generation hypothesis. *Journal of affective disorders* 2007; **99**(1): 147-54.
9. Snyder HR, Hankin BL. Spiraling out of control: Stress generation and subsequent rumination mediate the link between poorer cognitive control and internalizing psychopathology. *Clinical Psychological Science* 2016; **4**(6): 1047-64.
