# Supplementary material for: Stress sensitization among severely neglected children and protection by social enrichment
Source: Nat Commun. 2019 Dec 18;10:5771. doi: 10.1038/s41467-019-13622-3 (PMC6920417; doi:10.1038/s41467-019-13622-3)
Supplement: Supplementary file 3 — Reporting Summary [file 41467_2019_13622_MOESM3_ESM.pdf]

nature research

Corresponding author(s): DBPR NCOMMS-19-03981A

Last updated by author(s): Oct 17, 2019

Reporting Summary

Nature Research wishes to improve the reproducibility of the work that we publish. This form provides structure for consistency and transparency in reporting. For further information on Nature Research policies, see Authors & Referees and the Editorial Policy Checklist .

Statistics

For all statistical analyses, confirm that the following items are present in the figure legend, table legend, main text, or Methods section.

n/a	Confirmed	
☐	☒	The exact sample size (*n*) for each experimental group/condition, given as a discrete number and unit of measurement
☐	☒	A statement on whether measurements were taken from distinct samples or whether the same sample was measured repeatedly
☐	☒	The statistical test(s) used AND whether they are one- or two-sided<br>*Only common tests should be described solely by name; describe more complex techniques in the Methods section.*
☒	☐	A description of all covariates tested
☐	☐	A description of any assumptions or corrections, such as tests of normality and adjustment for multiple comparisons
☐	☒	A full description of the statistical parameters including central tendency (e.g. means) or other basic estimates (e.g. regression coefficient) AND variation (e.g. standard deviation) or associated estimates of uncertainty (e.g. confidence intervals)
☒	☐	For null hypothesis testing, the test statistic (e.g. *F*, *t*, *r*) with confidence intervals, effect sizes, degrees of freedom and *P* value noted<br>*Give P values as exact values whenever suitable.*
☒	☐	For Bayesian analysis, information on the choice of priors and Markov chain Monte Carlo settings
☒	☐	For hierarchical and complex designs, identification of the appropriate level for tests and full reporting of outcomes
☒	☐	Estimates of effect sizes (e.g. Cohen's *d*, Pearson's *r*), indicating how they were calculated

Our web collection on statistics for biologists contains articles on many of the points above.

Software and code

Policy information about availability of computer code

Data collection	Data were collected and are stored in RedCap.
Data analysis	The primary analyses were carried out in SPSS version 21. The code as been provided to the reviewers and editor for peer review.

For manuscripts utilizing custom algorithms or software that are central to the research but not yet described in published literature, software must be made available to editors/reviewers. We strongly encourage code deposition in a community repository (e.g. GitHub). See the Nature Research guidelines for submitting code & software for further information.

Data

Policy information about availability of data

All manuscripts must include a data availability statement. This statement should provide the following information, where applicable:

- Accession codes, unique identifiers, or web links for publicly available datasets
- A list of figures that have associated raw data
- A description of any restrictions on data availability

These data are not currently available for public use. Please direct any communications concerning data availability to the corresponding author.

Field-specific reporting

Please select the one below that is the best fit for your research. If you are not sure, read the appropriate sections before making your selection.

☐ Life sciences ☒ Behavioural & social sciences ☐ Ecological, evolutionary & environmental sciences

For a reference copy of the document with all sections, see nature.com/documents/nr-reporting-summary-flat.pdf

Behavioural & social sciences study design

All studies must disclose on these points even when the disclosure is negative.

Study description	Quantitative, longitudinal randomized controlled trial (RCT).
Research sample	Adolescents reared in institutions in Bucharest, Romania. This sample is not representative of the general population, but a comparison sample of never-institutionalized children who was matched on demographic characteristics was also recruited. Examining the effects of institutional deprivation on later stress and psychopathology necessitated using this uniquely vulnerable group of children.
Sampling strategy	Children were recruited from institutions across Bucharest, Romania. Access to the institutionalized children was granted through agreements with the Directorates of Child Protection in Romania. To enhance representativeness and bolster the participant pool, agreements were made with child protection authorities that were responsible for all of the young children in Bucharest, six in total. These permitted screening, identification, and placement into foster families (see Zeanah et al., 2003 for an in depth description).
Data collection	At multiple assessment time points, a trained researcher collected multiple types of data including self-report measures, observational data, standardized assessments, and biological measures (EEG, MRI, physiological recordings). Often more than one research assistant was required to assist with data collection. The researchers were not blind to the group the child belonged to.
Timing	The study began in April 2001, and the most recent (age 16) follow-up began in January 2015 and is ongoing.
Data exclusions	At the outset of the study, following the initial screen, 51 children (out of 187 total) were excluded on medical grounds, including fetal alcohol syndrome, microcephaly, Down syndrome, and related conditions. No additional data were excluded from this analysis.
Non-participation	Several participants have dropped out or experienced placement changes over time. This information is provided in the CONSORT flow diagram.
Randomization	Of the 136 children who met the inclusion criteria, half (n = 68) were randomly assigned to leave the institutions for foster care, and the other half (n = 68) were randomly assigned to the care as usual condition by drawing names from a hat.

Reporting for specific materials, systems and methods

We require information from authors about some types of materials, experimental systems and methods used in many studies. Here, indicate whether each material, system or method listed is relevant to your study. If you are not sure if a list item applies to your research, read the appropriate section before selecting a response.

Materials & experimental systems

n/a	Involved in the study	
☒	☐	Antibodies
☒	☐	Eukaryotic cell lines
☒	☐	Palaeontology
☒	☐	Animals and other organisms
☐	☒	Human research participants
☐	☒	Clinical data

Methods

n/a	Involved in the study	
☒	☐	ChIP-seq
☒	☐	Flow cytometry
☒	☐	MRI-based neuroimaging

Human research participants

Policy information about studies involving human research participants

Population characteristics	At age 12, the time point that stressful life events were measured, there was an equal number of males and females (50% of each). At age 12, 39% of the ever-institutionalized children met criteria for a psychiatric diagnosis compared to only 16% of the never-institutionalized children. Also, 25% of institutionally-reared children met criteria for an externalizing disorder compared to only 4% of never-institutionalized children (Humphreys et al., 2015). The current study focuses not on diagnoses, but on a dimensional score of externalizing problems at age 16 years.
Recruitment	As above.
Ethics oversight	Boston Children's Hospital; Tulane University; University of Maryland, College Park; Commissions on Child Protection in Bucharest; the Romanian Ministry of Health

Note that full information on the approval of the study protocol must also be provided in the manuscript.

Clinical data

Policy information about clinical studies
All manuscripts should comply with the ICMJE guidelines for publication of clinical research and a completed CONSORT checklist must be included with all submissions.

Clinical trial registration	NCT00747396
Study protocol	On the project website: http://www.bucharestearlyinterventionproject.org/ and in Zeanah, Nelson, Fox, Smyke, Marshall, Parker, & Koga (2003; Development and Psychopathology).
Data collection	Data collection has taken place in Bucharest, Romania. The study began in April 2001, and the most recent (age 16) follow-up began in January 2015 and is ongoing.
Outcomes	Primary outcome measures included psychopathology, including externalizing problems, the focus of the current study. Secondary outcomes included, among others, the life events measure used as the predictor in the current study.
